# Psychological predictors of change in the number of musculoskeletal pain sites among Norwegian employees: a prospective study

**DOI:** 10.1186/s12891-017-1503-7

**Published:** 2017-04-04

**Authors:** Jan Olav Christensen, Sissel Johansen, Stein Knardahl

**Affiliations:** 1 Department of Work Psychology and Physiology, The National Institute of Occupational Health, Oslo, Norway; 2 Oslo and Akershus University College of Applied Sciences, Faculty of Social Sciences, Department of Social Work, Child Welfare and Social Policy, Oslo, Norway

## Abstract

**Background:**

The pathogenesis of syndromes of widespread musculoskeletal pain remains an enigma. The present study sought to determine if *psychological states*, *job satisfaction*, *pain intensity*, and *sleep problems* contributed to the spread and decline of the number of musculoskeletal pains.

**Methods:**

A sample of 2989 Norwegian employees completed a questionnaire at baseline and follow-up 2 years later. Data were analyzed with multinomial and ordinal logistic regression analyses to determine effects on *direction* and *degree* of change of number of pain sites (NPS).

**Results:**

After adjustment for sex, age, skill level, and number of pain sites at baseline, *increases* in the number of pain sites from baseline to follow-up were predicted by *emotional exhaustion*, *mental distress*, *having little surplus*, *feeling down and sad*, *sleep disturbances*, and intensity of *headache. Decreases* were predicted by low levels of *emotional exhaustion*, *mental distress*, *sleep disturbances*, *restlessness*, and lower intensity of headache, neck pain, shoulder pain, and back pain. Higher numbers of pain sites at baseline were associated with reduction of number of pain sites and lower likelihood of spread. Some factors that did not predict *whether* decrease or increase occurred were nevertheless associated with the *degree* of decrease (depression, anxiety, having surplus, self-efficacy) or increase (anxiety).

**Conclusions:**

Several psychological and physiological factors predicted change in the number of pain sites. There is a need for further investigations to identify possible mechanisms by which psychological and behavioral factors propagate the spread of pain.

**Electronic supplementary material:**

The online version of this article (doi:10.1186/s12891-017-1503-7) contains supplementary material, which is available to authorized users.

## Background

The incidence of chronic musculoskeletal pain in the general population is high [1, 2], and musculoskeletal disorders are the most prevalent health complaints among European employees [3]. Among individuals with musculoskeletal pain, reporting pain in five or more sites has been found to be more common than reporting pain in one site only [4]. Multi-site pain has been found to predict sickness absence [5] and work ability [6]. It has been suggested that counting the concurrent number of pain sites (NPS) may serve as a method of identifying risk of disability [6, 7].

Hypotheses of the pathogenesis of spread of pain seem to focus mainly on (i) alterations of nociceptive pathways of the central nervous system, e.g. “central sensitization”, the definition of which is rather imprecise [8, 9], or (ii) psychological mechanisms like attention to somatic sensations (e.g. perceptual amplification [10]). The objective of the present study was to determine the contribution of psychological factors commonly associated with pain to subsequent spread or reduction of the number of musculoskeletal pain sites reported by employees in a working population.

Mental distress, anxiety, and depression are associated with chronic pain (see e.g. [11]), and depressive symptoms seem to contribute to the development and persistence of chronic musculoskeletal pain [12, 13]. Therefore, we sought to determine the contribution from several aspects of emotional state to changes in NPS. Catastrophizing seems related to some chronic pain states [14]. We sought to determine if the opposite of pessimistic attitudes may play a role, hence we included optimism and general self-efficacy. Sleep disturbance is one of the most prevalent co-morbid problems among pain patients [15, 16] and has been found to contribute to both onset and amplification of pain [17]. Sleep restriction may increase risk of next-day pain [18]. A 14-year follow-up study demonstrated that initial multi-site pain, sleep quality, sex, age and educational level were risk factors for an increased number of pain in a Norwegian county population [19]. We sought to elucidate the role of sleep disturbances and tiredness for spread of pain. Finally, we sought to determine if the intensity of pain in specific sites increased the risk of more widespread pain.

In short, the present study examined a large sample of Norwegian working individuals to determine whether psychological states and traits, sleep problems, and pain intensity predicted increases and decreases in the number of painful anatomic sites over a 2 years period.

## Methods

### Design

The study was a two-wave prospective full-panel study. All variables were measured by web-based self-report questionnaires at baseline and follow-up 2 years later.

### Subjects

Subjects were recruited from Norwegian companies that volunteered to participate. Baseline data were gathered from 2004 to 2011, follow-up data from 2006 to 2013. The follow-up period was approximately 2 years for all respondents (mean: 24 months, SD: 2.8 months, range: 18–36 months).

The current study was part of a larger project measuring a range of work- and health factors. Sixty-three organizations were included in this study, with a variety of job types from the private and public sector (see Table 1). The companies received reports and presentations of results as a tool for organizational development in return for participation and making the data available for research. For more detailed description of procedures, see [20].

**Table 1:** Baseline characteristics of all invited subjects, responders at T1^a^, responders at both T1 and T2^b^, and the final prospective sample after exclusion^c^

	All invited (*N* = 10274)	Responders at T1 (*n* = 6198)	Association with *non-response* at T1		Responders at both T1 and T2 (*n* = 4204)	Association with dropout after T1^d^		Prospective sample after exclusion (*n* = 2989)
	*N* (%)	*N* (%)	OR	95% CI	*N* (%)	OR	95% CI	*N* (%)
Sex								
Male	3852 (37.5)	2405 (38.8)	ref	-	1681 (40.0)	ref	-	1161 (38.8)
Female	6422 (62.5)	3793 (61.2)	1.16	[1.07–1.26]**	2523 (60.0)	1.02	[0.89–1.17]	1828 (61.2)
Age								
< 30	796 (7.7)	451 (7.3)	ref	-	269 (6.4)	ref	-	196 (6.6)
30–39	2519 (24.5)	1556 (25.1)	0.81	[0.69–0.95]*	1048 (24.9)	0.82	[0.63–1.07]	733 (24.5)
40–49	3118 (30.3)	1933 (31.2)	0.80	[0.68–0.94]**	1330 (31.6)	0.72	[0.55–.95]*	970 (32.5)
50–59	2973 (28.9)	1775 (28.6)	0.89	[0.76–1.04]	1230 (29.3)	0.74	[0.56–.97]*	870 (29.1)
> 59	868 (8.4)	483 (7.8)	1.06	[0.87–1.28]	327 (7.8)	0.83	[0.60–1.17]	220 (7.4)
Marital status								
Not married		618 (13.1)			444 (13.8)	ref	-	309 (13.5)
Married		2893 (61.2)			1982 (61.6)	1.21	[0.99–1.49]	1410 (61.6)
Cohabiting		764 (16.2)			499 (15.5)	1.32	[1.04–1.67]	370 (16.2)
Widowed		62 (1.3)			40 (1.2)	1.40	[0.78–2.51]	26 (1.1)
Divorced		313 (6.6)			203 (6.3)	1.34	[0.98–1.82]	137 (6.0)
Separated		75 (1.6)			47 (1.5)	1.47	[0.88–2.46]	38 (1.7)
Missing data		1473 -			989 -	-	-	699 -
Occupation								
Professionals		1757 (29.0)			1283 (31.3)	ref	-	904 (30.9)
Armed forces and unspecified		32 (0.5)			20 (0.5)	1.78	[0.83–3.82]	15 (0.5)
Legislators, senior officials and managers		587 (9.7)			464 (11.3)	0.81	[0.62–1.05]	307 (10.5)
Technicians and associate professionals		2040 (33.7)			1323 (32.3)	1.66	[1.41–1.95]**	954 (32.6)
Clerks		501 (8.3)			313 (7.6)	1.73	[1.36–2.21]**	244 (8.3)
Service workers and shop and market sales workers		979 (16.2)			605 (14.8)	1.70	[1.40–2.05]**	443 (15.2)
Skilled agricultural and fishery workers		2 (0.0)			1 (0.0)	2.85	[0.17–46.49]	0 (0.0)
Craft and related trades workers		67 (1.1)			43 (1.0)	2.11	[1.19–3.75]*	29 (1.0)
Plant and machine operators and assemblers		8 (0.1)			3 (0.1)	4.86	[0.81–29.35]	2 (0.1)
Elementary occupations		76 (1.3)			45 (1.1)	2.10	[1.26–3.50]**	26 (0.9)
Missing data		149 -			104 -	-	-	65 -
Skill level								
> 15 years		1757 (29.0)			1283 (31.3)	ref	-	904 (30.9)
13–15 years		2040 (33.7)			1323 (32.3)	1.66	[1.41–1.95]**	954 (32.6)
10–12 years		1557 (25.7)			965 (23.5)	1.73	[1.46–2.05]**	718 (24.6)
< 10 years		76 (1.3)			45 (1.1)	2.10	[1.26–3.50]**	26 (0.9)
Unspecified		619 (10.2)			484 (11.8)	0.86	[0.67–1.10]	322 (11.0)
Missing data		149 -			104 -	-	-	65 -
Number of pain sites at T1								
0		1446 (23.3)			1001 (23.8)	ref	-	-
1		1359 (21.9)			962 (22.9)	0.86	[0.71–1.04]	962 (32.2)
2		1299 (21.0)			884 (21.0)	0.99	[0.82–1.20]	884 (29.6)
3		1082 (17.5)			706 (16.8)	1.13	[0.92–1.38]	706 (23.6)
4		680 (11.0)			437 (10.4)	1.13	[0.90–1.41]	437 (14.6)
5		332 (5.4)			214 (5.1)	1.21	[0.90–1.63]	-

*p<0.05, **p<0.01

^a^Response at T1 was defined as having completed all five pain items at T1

^b^Response at both T1 and T2 was defined as having completed all pain items at both T1 and T2

^c^The final prospective sample comprised employees that participated at both T1 and T2 and that reported at least one but no more than four pain sites at T1

^d^Association with dropout was computed among T1 responders by multivariable logistic regressions with all predictors included simultaneously, except skill level and occupation which were entered separately since they were different categorizations of the same variable. ‘’Dropout” was defined as responding at T1, but not volunteering information about all five studied pain sites at T2

Employees and management were first informed at the organizational level before all employees received a letter with an invitation and information about the survey. The letter included a personal access code to the web-questionnaire or a paper version of the questionnaire with a pre-stamped return envelope.

**Fig. 1:**
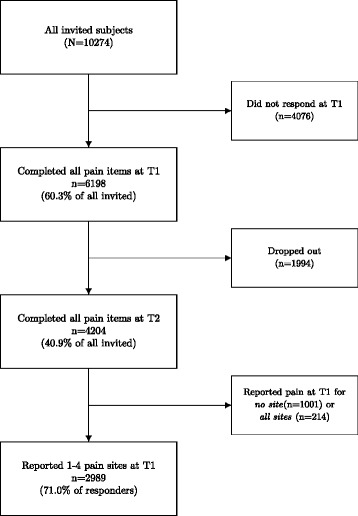
Overview of the study sample

Only respondents who answered whether they had pain or not in all five pain sites listed in the questionnaire, at both time points, were included. A total of 10274 employees were invited at both the first (T1) and the second survey (T2), of which 6198 (60.3%) answered all five pain region questions at T1. The primary interest of the study pertained to the course of existing pain problems. Therefore, respondents who reported no pain in any anatomic site at T1 were excluded from analyses of pain change direction, as this group’s report of pain at T2 may represent *onset* and not *spread* of pain. In addition, a decrease in the number of pain sites would not be possible for this group. Likewise, subjects reporting pain in all sites at T1 were excluded, as pain spread would not be possible for this group. Thus, the final main sample comprised 2989 subjects that reported 1–4 pain sites at T1 *and* completed all five pain items at both time points (see Fig. 1 and Table 2).

**Table 2:** Separate multinomial logistic regressions relating psychological factors and pain intensities at T1 to number of pain sites (NPS) *decrease* (NPS T1 > NPS T2) and *increase* (NPS T1 < NPS T2) compared with reporting a *stable* number of pain sites (NPS T1 = NPS T2)

T1 Predictor	N	Decrease NPS (T1 > T2)		Increase NPS (T1 < T2)	
		OR	95% CI	OR	95% CI
Emotional exhaustion	2644	0.81	[0.69–0.95] **	1.34	[1.15–1.57] ***
Mental distress (HSCL)	2752	0.69	[0.54–0.89] ***	1.64	[1.28–2.10] ***
Depression (single item)	2912	0.90	[0.76–1.05]	1.16	[0.99–1.37]
Anxiety (single item)	2909	0.86	[0.66–1.11]	1.09	[0.84–1.41]
Psychological well-being					
*“Had a lot of surplus”*	2814	1.02	[0.95–1.10]	0.87	[0.80–0.95] ***
*“Felt down and sad”*	2808	0.98	[0.88–1.09]	1.18	[1.06–1.31] ***
Dispositional optimism	2858	1.08	[0.94–1.25]	0.94	[0.81–1.09]
General self-efficacy	2862	0.97	[0.82–1.14]	1.05	[0.88–1.26]
Job satisfaction	2608	1.05	[0.90–1.22]	0.97	[0.82–1.15]
Sleep disturbance	2801	0.87	[0.79–0.96] ***	1.19	[1.07–1.31] ***
Tiredness	2919	0.82	[0.73–0.93] ***	1.24	[1.09–1.40] ***
Restlessness	2915	0.80	[0.69–0.92] ***	1.08	[0.93–1.25]
Pain intensity					
*Headache*	2921	0.81	[0.72–0.91] ***	1.17	[1.04–1.32] **
*Neck pain*	2924	0.64	[0.56–0.73] ***	1.02	[0.89–1.17]
*Shoulder pain*	2924	0.78	[0.68–0.89] ***	0.98	[0.85–1.12]
*Arm pain*	2924	1.09	[0.95–1.25]	0.97	[0.83–1.14]
*Leg pain*	2924	1.06	[0.93–1.21]	1.03	[0.88–1.19]
*Back pain*	2924	0.85	[0.76–0.96] **	1.06	[0.93–1.20]
Number of pain sites at T1	2924	1.33	[1.22–1.45] ***	0.79	[0.72–0.87] ***

*p < 0.05, **p < 0.01, ***p < 0.001

*Note:* Subjects that reported either no pain sites or five pain sites at T1 were excluded since they could not experience decrease or increase of NPS, correspondingly. All regressions were adjusted for sex, age group, skill level, and T1 NPS

### Ethics

The study was approved by the Norwegian Data Inspectorate and the Regional Committees for Medical and Health Research Ethics (REK) in Norway and conducted in accordance with the World Medical Association Declaration of Helsinki. All study participants provided informed consent. The consent procedure was approved by the Data Inspectorate of Norway and REK. Data were stored and analyzed in accordance with procedures designed to ensure the anonymity of participants.

### Outcome: change of number of pain sites

Musculoskeletal pain was measured by reported intensity of pain in five anatomic sites during 4 weeks prior to answering the questionnaire; neck, shoulder, back, arm, and leg [21]. Each question was phrased: “Have you been troubled by… (e.g. neck pain) the last 4 weeks? Response alternatives were: 1) “not troubled”, 2) “a little troubled”, 3) “rather intensely troubled” and 4)” very intensely troubled”. In Norwegian, the wording “troubled by” is a common way of expressing symptoms. Number of pain sites scales for baseline (NPS T1) and follow-up (NPS T2) were constructed. First, each question (i.e. region) was dichotomized to “0 = not troubled” and “1 = troubled”. Then the NPS scales were constructed by summing these five items.

In order to capture the *direction* as well as the *degree* of change in NPS different variables were constructed based on the difference between T1 and T2 NPS.

First, a three-category variable was constructed to reflect *stability*, *decrease*, and *increase* of NPS from T1 to T2. Stability was operationalized as reporting the same number of pain sites at T1 and T2. Decrease was defined as reporting at least one pain site less at T2, and increase as reporting at least one pain site more at T2 than at T1. No inherent order was assumed among these three categories, hence the variable was treated as nominal.

The degree of change was assessed separately for the three groups defined by the change direction variable. Thus, for those experiencing increase and decrease the degree of change was operationalized as the number of pain sites more or less at T2 than at T1. For those reporting the same number of pain sites at T1 and T2 the outcome variable was the number of pain sites that they reported.

### Predictors

*Pain intensity* of each musculoskeletal site was measured as described above. Additionally, *headache, tiredness, restlessness, depression*, and *anxiety* were measured with equivalent single items and the same response categories.

*Emotional exhaustion* was measured with six items from the Copenhagen Burnout Inventory [22]; how often… “do you feel tired?”, “are you physically exhausted?”, “are you emotionally exhausted?”, “do you think ‘I can’t stand anymore’?”, “do you feel worn out”, and “do you feel weak and susceptible to diseases”. Response alternatives were “1 = never/almost never”, “2 = a couple of times per month”, “3 = once or twice a week”, “4 = three to four times a week”, and “5 = (almost) every day”. Items were combined into one scale (α = 0,83). The average score was estimated and the variable was recoded: “1.00-1.50” set to “1”, “1.51–2.50” set to “2”, “2.51–3.50” set to “3”, “3.51–4.50” set to “4” and “4.51–5.00” set to “5”.

*Psychological well-being* was measured with two single items: “The last 4 weeks, how often have you…..” a)” had a lot of surplus” and b) “felt down and sad”. Response categories were “1 = all the time”, “2 = almost all the time”, “3 = much of the time”, “4 = some of the time”, “5 = a bit of the time”, and “6 = not at all”. To aid interpretation, the items were reversed so high levels reflected high perceived surplus and feelings of sadness.

*Sleep disturbance* was measured with two separate items: “Have you noticed the following problems the last 4 weeks”: a) “difficulties with sleeping” and b)” disturbed/restless sleep”. Response categories were “1 = zero”, “2 = 1–3 times per month”, “3 = 1–2 times a week”, “4 = 3–5 times a week” and “5 = 6–7 times a week”. These items were combined into one scale (α = 0,82). The average score was estimated, and the variable recoded for analyses with categorical predictors: “1.00–1.50” set to “1”, “1.51–2.50” set to “2”, “2.51–3.50” set to “3”, “3.51–4.50” set to “4”, and”4,51–5,00” set to “5”.

*Global job satisfaction* was assessed with one question: “All things considered, how satisfied are you with your job?” with alternatives “1 = very dissatisfied”, “2 = dissatisfied”, “3 = satisfied” and “4 = very satisfied”. The scale was reversed for the analyses.

*Optimism* was measured with three items from the “Revised Life Orientation Test (LOT-R)” [23]: “In uncertain times, I usually expect the best”, “I hardly ever expect things to go my way” (reversed item), “overall, I expect more good things to happen to me than bad”. Response categories were “1 = strongly disagree”, “2 = disagree”, “3 = neutral”, “4 = agree” and “5 = strongly agree”. Items were combined into one scale (α = 0,61). The average score was estimated, and the variable recoded: “1.00–1.50” set to “1”, “1.51–2.50” set to “2”, “2.51–3.50” set to “3”, “3.51–4.50” set to “4”, and”4,51–5,00” set to “5”.

Due to a low number of employees reporting “strongly disagree” and “disagree” the two first categories of the categorized measure were collapsed.

*General self-efficacy* was measured with three items from the instrument developed by Schwarzer and Jerusalem [24]: “I can solve most problems if I just make an effort”, “If I am in trouble, I usually find a solution”, “No matter what happens; I am usually capable of handling it”. Alternatives were “1 = strongly disagree”, “2 = disagree”, “3 = neutral”, “4 = agree” and “5 = strongly agree”. The items were combined into one scale (α = 0,83). Since very few employees reported low self-efficacy the scale was reversed before analyses to maximize statistical power in categorical analyses with the first category as reference.

*Mental distress* during the last week was measured by ten items from the Hopkins Symptom Checklist (HSCL-10) [25]. Example items are “Feeling tense or keyed up” and “A feeling of hopelessness about the future”, rated on a four-point scale ranging from “1 = have not experienced it” to “4 = very much”. HCSL-10 has been demonstrated to be a valid and reliable measure of psychological distress [26]. Items were combined into one scale (α = 0,86). The average score was estimated and the variable recoded: “1.00–1.50” set to “1”, “1.51–2.50” set to “2”, “2.51–3.50” set to “3”, “3.51–4.00” set to “4”. Due to a small number of subjects in the last category, “3” and “4” were collapsed.

*Age, sex* and *skill level* were included in all multivariable regressions as possible confounders. *Age* was classified into age groups: “<30”, “30–39”, “40–49”, “50–59” and “>59”. *Skill levels* were derived from information reported by the companies. Classifications were based on the International Standard Classification of Occupations (ISCO-88), which is a tool to organize jobs into groups according to the tasks and duties typically undertaken in the job. Educational level or equivalent working experience required for the job is also reflected by the ISCO-88, according to the International Standard Classification of Education (ISCED). *Skill level* categories thus denote occupations that normally require (1) = first or postgraduate university degree or college exams of similar level (>16 years of education); (2) = 1–3 years of college/university education (13–15 years); (3) = 1–3 years of secondary education (10–12 years); (4) = primary education (<9 years) and (5) = unspecified competence level (i.e. occupations with no formally required education).

### Statistical analyses

All statistical analyses were conducted with R, version 3.3.1 [27].

*Non-response analyses* were conducted by multivariable binary logistic regressions. The odds of non-response (i.e. failing to answer all pain items) at baseline were computed based on sex and age. Next, the risk of *attrition bias* was assessed by computing the odds of being a non-responder at the second survey among those who responded at T1. For the attrition analysis, sex, age, marital status, skill level, and number of pain sites were entered as predictors in a multivariable logistic regression.

In order to study the relationships of the included factors with direction of change of NPS, multinomial regressions were conducted to determine relationships between baseline predictors and NPS change. First, regressions were conducted separately for each predictor adjusted for age group, sex, skill level, and T1 NPS. Then, all predictors were entered into one regression to determine any unique associations between predictors and direction of change after mutual adjustment. The multinomial regressions expressed the effect of the predictors on the likelihood of experiencing pain increase or decline when compared with experiencing stability in NPS, controlled for sex, age, skill level and initial NPS.

The association of predictors with the *degree* of change in NPS was assessed by running regression separately for the groups that reported decrease and increase of NPS to predict the number of pain sites more or less at T2. Since the ΔNPS variable was skewed ordinal logistic regressions were conducted. Ordinal regression expresses the change in odds of having a higher level of the outcome (i.e. ΔNPS) per unit change of the predictor, assuming that the difference in odds is the same for all cut points of the outcome variable.

Regressions of NPS change direction were also conducted with predictors categorized, to reflect the effects of specific levels of the predictor. Likelihood ratio tests (LRTs) were computed to determine whether models with categorized predictors represented a statistically significant improvement over the corresponding models with continuous predictors, which would suggest the presence of non-linear effects such as e.g. threshold effects.

## Results

At baseline, 23.3% of subjects reported no pain, 21.9% reported one pain site, 21% reported two sites, 17.5% three sites, 11% four sites, and 5.4% reported five pain sites (Table 1). Spread of pain, i.e. reporting pain in at least one additional anatomic site at T2 compared to T1, was reported by 31.6% (*n* = 1329) if including subjects that reported no pain at T1, which may include subjects experiencing onset as well as spread of pain. Among those reporting at least one pain site at T1, but not five pain sites, 29.2% (*n* = 874) experienced an increase in NPS (Additional file 1: Table S1). A decrease in NPS was observed for 29.1% (*n* = 1224) of the full sample, and for 37.2% (*n* = 1112) of those reporting 1–4 pain sites at T1 (Additional file 1: Table S1).

### Dropout and attrition analyes

Among invited subjects (*N* = 10274), non-response analyses showed that subjects aged 30–39 and 40–49 were more likely than others to return the questionnaire with completed information about all five pain sites at the first survey (ORs of non-response: 0.81, 95% CI 0.69–0.95 and OR 0.80, 95% CI 0.68–0.94, respectively) (Table 1). Women were less likely to respond than men (OR 1.16 of non-response, 95% CI 1.07–1.26) (Table 1).

Attrition analyses among the 6198 who responded to the first survey revealed that age groups 40–49 and 50–59 were less likely to drop out (i.e. more likely to also return information at T2), while those employed as technicians and associate professionals, clerks, service workers and shop and market sales workers, craft and related trades workers, and elementary occupations were more likely to drop out. Employees with the highest skill level, i.e. jobs reflecting more than 15 years of education, were least likely to drop out (Table 1).

### Adjusted own effects

After adjustment for sex, age, skill level and T1 NPS, decreases in NPS were less likely for those reporting higher levels of emotional exhaustion (OR 0.81 95% CI 0.69–0.95), mental distress (OR 0.69, 95% CI 0.54–0.89), sleep disturbance (OR 0.87, 95% CI 0.79–0.96), tiredness (OR 0.82, 95% CI 0.73–0.93), restlessness (OR 0.80, 95% CI 0.69–0.92), headache intensity (OR 0.69, 95% CI 0.54–0.89), neck pain intensity (OR 0.64, 95% CI 0.56–0.73), shoulder pain intensity (OR 0.78, 95% CI 0.68–0.89), and back pain intensity (OR 0.85, 95% CI 0.76–0.96).

Increased NPS (spread of pain) from baseline to follow-up was more likely for higher levels of emotional exhaustion (OR 1.34, 95% CI 1.15–1.57), mental distress (OR 1.64, 95% CI 1.28–2.10), feeling down and sad (OR 1.18, 95% CI 1.06–1.31), sleep disturbance (OR 1.19, 95% CI 1.07–1.31), tiredness (OR 1.24, 95% CI 1.09–1.40), and headache intensity (OR 1.17, 95% CI 1.04–1.32), and less likely for higher levels of surplus (OR 0.87, 95% CI 0.80–0.95). A higher number of pain sites at T1 was associated with higher likelihood of decrease (OR 1.33, 95% CI 1.22–1.45) and lower likelihood of increase (OR 0.79, 95% CI 0.72–0.87). Likelihood ratio tests suggested that entering predictors as categorical improved the models for the intensity of headache, shoulder pain, leg pain, back pain, and arm pain (see Additional file 1: Table S4). For pain in the leg, back, and arm the likelihood of decrease in the number of pain sites was elevated for subjects reporting light pain compared to no pain or moderate to severe pain, and for arm pain and leg pain, these statistically significant effects were not detected with the linear predictor, as can be seen in Table 2.

The analyses of the *degree of change* can be seen in Table 3. They revealed that among those who experienced increases of NPS, more severe increases were predicted by emotional exhaustion (OR 1.34, 95% CI 1.10–1.64), mental distress (OR 1.67, 95% CI 1.22–2.30), anxiety (OR 1.40, 95% CI 1.03–1.90), sleep disturbance (OR 1.29, 95% CI 1.14–1.47), and headache intensity (OR 1.24, 95% CI 1.06–1.46). On the other hand, having a lot of surplus predicted less severe increases for this group (OR 0.83, 95% CI 0.75–0.92). For subjects experiencing decreases, a slower rate of decrease was predicted by higher levels of emotional exhaustion (OR 0.64, 95% CI 0.51–0.80), mental distress (OR 0.45, 95% CI 0.31–0.65), depression (OR 0.64, 95% CI 0.51–0.80), anxiety (OR 0.45, 95% CI 0.29–0.67), lower general self-efficacy (OR 0.77, 95% CI 0.61–0.98), sleep disturbance (OR 0.80, 95% CI 0.70–0.92), tiredness (OR 0.81, 95% CI 0.68–0.96), restlessness (OR 0.76, 95% CI 0.62–0.93), headache intensity (OR 0.83, 95% CI 0.70–0.98), neck pain intensity (OR 0.67, 95% CI 0.55–0.81), and back pain intensity (OR 0.79, 95% CI 0.65–0.94). A higher rate of decrease was predicted by having more surplus (OR 1.21, 95% CI 1.08–1.35). A higher initial number of pain sites predicted a higher rate of decrease (OR 1.90, 95% CI 1.71–2.12) and lower rate of increase (OR 0.71, 95% CI 0.64–0.79). Table 3 also shows that all studied factors except general self-efficacy exhibited statistically significant associations with the number of pain sites reported by those subjects that reported the same number at both time points, with ORs ranging from 0.50, 95% CI 0.43–0.59 for job satisfaction to 9.15, 95% CI 7.85–10.71 for neck pain.

**Table 3:** Separate ordinal logistic regressions relating psychological factors and pain intensities at T1 to: 1) the number of pain sites among subjects reporting the same NPS at T1 and T2, 2) the number of pain sites *decreased* among subjects that reported a higher number of pain sites at T1, and 3) the number of pain sites *increased* among subjects that a higher number of pain sites at T2

Sub-sample:	Employees reporting *stable* number of pain sites (T1 NPS = T2 NPS)			Employees reporting *decreased* number of pain sites (T1 NPS > T2 NPS)			Employees reporting *increased* number of pain sites (T1 NPS < T2 NPS)		
Outcome variable:	*Number of pain sites*			*Number of pain sites decreased*			*Number of pain sites increased* (spread)		
T1 Predictor	N	OR	95% CI	N	OR	95% CI	N	OR	95% CI
Emotional exhaustion	1444	3.99	[3.38–4.73]***	1080	0.64	[0.51–0.80]**	1162	1.34	[1.10–1.64]**
Mental distress (HSCL)	1515	7.10	[5.47–9.24]***	1124	0.45	[0.31–0.65]**	1208	1.67	[1.22–2.30]**
Depression (single item)	1601	2.30	[1.95–2.72] ***	1194	0.64	[0.51–0.80]***	1286	0.99	[0.79–1.24]
Anxiety (single item)	1598	3.05	[2.33–4.00] ***	1192	0.45	[0.29–0.67]***	1288	1.40	[1.03–1.90]*
*Psychological well-being*									
*“Had a lot of surplus”*	1541	0.58	[0.53–0.62]***	1146	1.21	[1.08–1.35]**	1245	0.83	[0.75–0.92]**
*“Felt down and sad”*	1536	1.64	[1.47–1.84]***	1147	0.90	[0.78–1.04]	1242	1.09	[0.96–1.24]
Dispositional optimism	1578	0.60	[0.52–0.70] ***	1175	1.15	[0.93–1.41]	1255	0.85	[0.71–1.02]
General self-efficacy	1577	1.16	[0.97–1.38]	1177	0.77	[0.61–0.98]*	1263	1.15	[0.92–1.42]
Job satisfaction	1442	0.50	[0.43–0.59] ***	1068	1.19	[0.96–1.47]	1148	0.83	[0.68–1.03]
Sleep disturbance	1537	1.90	[1.73–2.10]***	1154	0.80	[0.70–0.92]**	1231	1.29	[1.14–1.47]**
Tiredness	1606	2.50	[2.21–2.82]***	1197	0.81	[0.68–0.96]*	1287	1.15	[0.98–1.35]
Restlessness	1604	2.12	[1.83–2.46]***	1194	0.76	[0.62–0.93]**	1289	1.20	[0.98–1.45]
*Pain intensity*									
*Headache*	1608	3.04	[2.67–3.46]***	1196	0.83	[0.70–0.98]*	1292	1.24	[1.06–1.46]**
*Neck pain*	1610	9.15	[7.85–10.71]***	1197	0.67	[0.55–0.81]**	1293	1.04	[0.86–2.35]
*Shoulder pain*	1610	8.70	[7.44–10.22]***	1197	0.89	[0.73–1.08]	1293	0.83	[0.66–1.03]
*Arm pain*	1610	6.01	[5.07–7.17]***	1197	0.94	[0.77–1.13]	1293	0.80	[0.59–1.05]
*Leg pain*	1610	4.47	[3.82–5.26]***	1197	0.89	[0.74–1.08]	1293	1.04	[0.82–1.32]
*Back pain*	1610	5.76	[5.01–6.64]***	1197	0.79	[0.65–0.94]**	1293	1.12	[0.93–1.35]
Number of pain sites	-	-	-	1197	1.90	[1.71–2.12]**	1293	0.71	[0.64–0.79]**

*p < 0.05, **p < 0.01, ***p < 0.001

*Note:* All regressions with decrease and increase as outcome were adjusted for sex, age group, skill level, and T1 NPS. For these regressions subjects that reported no pain sites at T1 were included in the increase group and subjects reporting five pain sites at T1 were included in the decrease group

### Mutually adjusted effects

Regressions adjusted for all other studied factors revealed that the following factors remained statistically significant predictors: having a lot of surplus (for decrease NPS: OR 0.87, 95% CI 0.78–0.97, for degree of increase: OR 0.84, 95% CI 0.73–0.97), *restlessness* (for decrease NPS: OR 0.77, 95% CI 0.62–0.96)*, sleep disturbance* (for degree of increase: OR 1.24, 95% CI 1.03–1.49)*, depression (for degree of increase: OR 0.69, 95% CI 0.48–0.98), neck pain* (for decrease NPS: OR 0.63, 95% CI 0.53–0.76, for degree of decrease: OR 0.61, 95% CI 0.47–0.79), *shoulder pain* (for decrease NPS: OR 0.79, 95% CI 0.66–0.93)*, back pain* (for decrease NPS: OR 0.78, 95% CI 0.66–0.92, for degree of decrease: OR 0.73, 95% CI 0.58–0.92) (Tables 4 and 5). Higher number of pain sites at T1 increased the likelihood of decrease (OR 2.04, 95% CI 1.69–2.47), decreased the likelihood of increase (OR 0.69, 95% CI 0.56–0.85), and increased the likelihood of faster decrease (OR 3.11, 95% CI 2.45–3.98). Among subjects with sTable NPS, anxiety, surplus, tiredness, restlessness, and the intensity of pain in the neck, shoulder, arm, leg, and back were associated with the number of pain sites reported (ranging from OR 0.51, 95% CI 0.29–0.88 for anxiety to OR 6.85, 95% CI 5.29–8.97 for leg pain intensity).

**Table 4:** Multivariable multinomial logistic regression with all predictors entered simultaneously and NPS decrease and increase as outcomes, with stable NPS as reference (*N* = 2199)

T1 Predictor	Decrease NPS		Increase NPS	
	OR	95% CI	OR	95% CI
Emotional exhaustion	0.92	[0.71–1.19]	1.00	[0.78–1.34]
Mental distress	0.87	[0.53–1.44]	1.50	[0.90–2.50]
*Depression (single item)*	1.20	[0.93–1.56]	0.95	[0.73–1.25]
*Anxiety (single item)*	1.14	[0.80–1.62]	0.95	[0.66–1.35]
*Psychological well-being*				
*“Had a lot of surplus”*	0.87	[0.78–0.97] **	0.94	[0.84–1.05]
*“Felt down and sad”*	1.06	[0.91–1.24]	1.07	[0.91–1.26]
Dispositional optimism	1.05	[0.87–1.27]	1.07	[0.88–1.30]
General self-efficacy	1.06	[0.85–1.31]	1.09	[0.87–1.37]
Job satisfaction (single item)	0.96	[0.80–1.15]	1.14	[0.93–1.39]
Sleep disturbance	0.96	[0.84–1.10]	1.07	[0.93–1.23]
Tiredness	0.86	[0.72–1.02]	0.99	[0.82–1.18]
Restlessness	0.77	[0.62–0.96] *	0.95	[0.76–1.18]
*Pain intensity*				
*Headache*	0.90	[0.77–1.04]	1.09	[0.94–1.26]
*Neck pain*	0.63	[0.53–0.76] ***	0.96	[0.81–1.15]
*Shoulder pain*	0.79	[0.66–0.93] **	1.07	[0.90–1.28]
*Leg pain*	0.90	[0.76–1.07]	1.01	[0.83–1.22]
*Back pain*	0.78	[0.66–0.92] ***	1.04	[0.88–1.23]
*Arm pain*	0.91	[0.77–1.09]	1.01	[0.83–1.22]
Number of pain sites	2.04	[1.69–2.47] ***	0.69	[0.56–0.85] ***

*p < 0.05, **p < 0.01, ***p < 0.001

*Note:* Subjects that reported either no pain sites or five pain sites at T1 were excluded since they could not experience decrease or increase of NPS, correspondingly. Adjustment was made for sex, age group, and skill level

**Table 5:** Multivariable ordinal logistic regressions with all psychological factors and pain intensities entered simultaneously as predictors of: 1) the number of pain sites among subjects reporting the same NPS at T1 and T2 (*N* = 1201), 2) the number of pain sites decreased among subjects that reported a higher number of pain sites at T1 (*N* = 909), and 3) the number of pain sites increased among subjects that a higher number of pain sites at T2 (*N* = 963)

Sub-sample:	Employees reporting *stable* number of pain sites (T1 NPS = T2 NPS)		Employees reporting *decreased* number of pain sites (T1 NPS > T2 NPS)		Employees reporting *increased* number of pain sites (T1 NPS < T2 NPS)	
Outcome variable:	*Number of pain sites*		*Number of pain sites decreased*		*Number of pain sites increased* (spread)	
T1 Predictor	OR	95% CI	OR	95% CI	OR	95% CI
Emotional exhaustion	1.00	[0.70–1.43]	0.94	[0.65–1.34]	0.99	[0.69–1.41]
Mental distress (HSCL)	0.88	[0.45–1.71]	0.57	[0.29–1.11]	1.16	[0.59–2.29]
Depression (single item)	1.07	[0.75–1.52]	0.74	[0.50–1.08]	0.69	[0.48–0.98] *
Anxiety (single item)	0.51	[0.29–0.88] *	0.63	[0.34–1.11]	1.48	[0.97–2.23]
*Psychological well-being*						
*“Had a lot of surplus”*	1.23	[1.06–1.43] **	1.04	[0.89–1.21]	0.84	[0.73–0.97] *
*“Felt down and sad”*	1.08	[0.88–1.32]	1.19	[0.97–1.45]	1.01	[0.84–1.22]
Dispositional optimism	0.93	[0.72–1.20]	0.89	[0.68–1.17]	0.85	[0.66–1.09]
General self-efficacy	1.03	[0.76–1.38]	0.89	[0.65–1.21]	0.95	[0.72–1.25]
Job satisfaction	1.03	[0.82–1.30]	0.87	[0.68–1.13]	0.96	[0.75–1.22]
Sleep disturbance	1.06	[0.88–1.27]	0.97	[0.80–1.17]	1.24	[1.03–1.49] *
Tiredness	1.28	[1.00–1.64] *	1.09	[0.84–1.41]	0.94	[0.74–1.18]
Restlessness	1.58	[1.16–2.14] **	1.07	[0.78–1.46]	0.90	[0.68–1.19]
*Pain intensity*						
*Headache*	0.92	[0.75–1.12]	0.93	[0.75–1.15]	1.14	[0.93–1.39]
*Neck pain*	5.33	[4.14–6.92] ***	0.61	[0.47–0.79] ***	0.80	[0.59–1.08]
*Shoulder pain*	6.78	[5.26–8.82] ***	0.89	[0.69–1.15]	0.75	[0.54–1.01]
*Arm pain*	6.33	[4.94–8.19] ***	0.89	[0.71–1.12]	1.02	[0.72–1.42]
*Leg pain*	6.85	[5.29–8.97] ***	0.85	[0.67–1.07]	0.79	[0.58–1.05]
*Back pain*	5.85	[4.61–7.48] ***	0.73	[0.58–0.92] **	0.70	[0.46–1.02]
Number of pain sites	-	-	3.11	[2.45–3.98] ***	0.86	[0.62–1.19]

*p < 0.05, **p < 0.01, ***p < 0.001

*Note:* The regressions with decrease and increase as outcome were adjusted for sex, age group, skill level, and T1 NPS

## Discussion

The present study supported the role of several psychological and physiological factors as influences on spread of pain and alleviation of multi-site pain. Emotional exhaustion, mental distress, sleep disturbance, tiredness and headache intensity appeared to affect the probabilities of both increased and decreased number of pain sites over 2 years. Restlessness, neck pain intensity, shoulder pain intensity, and back pain intensity seemed to influence decreases in NPS, while the experience of having a lot of surplus or feeling down and sad appeared to mainly influence the likelihood of spread of pain, by making it less likely and more likely, respectively. Moreover, depression and anxiety did not seem to directly influence whether or not decrease or increase happened, but were nevertheless related to the rate at which it did. The initial number of reported pain sites predicted both pain decrease and increase.

Limited knowledge exists about mechanisms of development of multisite musculoskeletal pain. We have not found longitudinal studies of psychological factors as predictors of spread and decline of musculoskeletal pain. Associations of headache and sleep disturbance with musculoskeletal pain have been reported, but the causal direction remains uncertain (see e.g. [16]). Our results indicate that headache plays a significant role in spread of musculoskeletal pain. Also, more intense back- or neck pain were robustly associated with subsequent change in NPS.

In a cohort study by Paananen and coworkers [28], short sleep duration (<7 h/day) predicted persistence of multiple pain sites over a 2-years period among adolescents, and good quality sleep has been found to predict positive musculoskeletal health, i.e. having no pain and/or alleviation of musculoskeletal pain [1, 29]. Although sleep problems are commonly comorbid to musculoskeletal pain [30, 31], a recent study by Anderson and colleagues [32] did not find that sleep disturbances predicted pain in fibromyalgia patients. However, the present study found that sleep disturbance and tiredness were significant predictors of NPS development [15, 33, 34]. 

Several psychological factors exhibited “adjusted own effects” on NPS change. Congruent with these findings, Mundal et al. [35] observed in a recent population-based study comprising 19000 individuals of a Norwegian county that both depression, anxiety, and sleep disturbances exhibited robust associations with the onset of chronic widespread pain over a period of 11 years.

However, in the current study none of the psychological factors predicted NPS change in the mutually adjusted analysis. For instance, *emotional exhaustion* and *mental distress* exhibited statistically non-significant effects in the final, fully adjusted model. Although associations between chronic pain and psychological distress, anxiety and depression are thoroughly documented (see e.g. review by Manchikanti et al. [11]) the causal status of this relationship is not resolved. One study by Estlander and colleagues [36] found that psychological distress, depression and self-efficacy beliefs did not predict changes in pain frequency. The fact that a few predictors remained statistically significant in the final, mutually adjusted analyses of the current study convincingly may suggest a crucial role for these factors in the development of musculoskeletal pain conditions.

However, great caution should be exercised in inferring substantive non-significance for *statistically non-significant* factors in those analyses. There are many possible explanations for the loss of association or statistical significance for some predictors when entered into multivariable analysis with other predictors, confounding being only one. Certainly, the possibility remains that some predictors are influenced by other predictors in the model. Several of the included predictors were correlated, and the mutually adjusted regression represented an attempt to uncover “core predictors”. However, in some instances the results may be hard to interpret. For instance, Table 4 suggests that having a lot of surplus lowers the likelihood of decrease in NPS, in contrast to Table 2, where it is associated with lowered odds of NPS increase. Thus it seems particularly pertinent to point out that the effect in Table 4 is conditional on effects of many conceptually similar factors as well as possible mediators of the relationship of interest. For instance, sleep problems is one indicator of mental distress incorporated as an item in the HSCL-10, the measure of psychological distress of the current study. The possibility that e.g. sleep problems represent an important *mediating mechanism* between several of the other independent factors and pain site development must be considered a viable explanation.

Back- and neck pain intensity at baseline predicted an increase in the number of pain sites from baseline to follow-up. Kamaleri and coworkers [37] found that multi-site pain at baseline was the most important predictor for an increase in number of pain sites 14 years later. Nevertheless, they also found that number of pain sites tended to remain stable over time, a finding supported by Gummesson and coworkers [38]. Several previous studies have reported that co-occurring pain in multiple musculoskeletal sites is common in working and patient populations [31, 39, 40]. The present study found that higher numbers of pain sites at baseline were associated with lowered risk of an increase in number of pain sites from baseline to follow-up. This may be partly due to regression towards the mean. Another possible explanation is that the 2-years span between baseline and follow-up is too short to detect spread of pain related to baseline pain. Although the present study showed that 29.2% experienced pain spread, for some subjects the increase may reflect fluctuating pain rather than the development of a condition characterized by multiple pain sites. Nevertheless, the current results support the notion that the *intensity* of current pain is a more reliable marker of risk of spread than the *number* of pre-existing pain sites.

There is strong evidence that specific psychosocial work factors and job satisfaction predict pain (e.g. [41–44]). Job satisfaction was not associated with pain spread in the present study. Intensity, onset, and spread of pain are, however, different endpoints and the pathogeneses of pain *onset* and *spread* may differ. Moreover, employees with high job satisfaction did indeed report fewer pain sites among subjects with stable NPS (see Table 3). Such results are of course equivalent to cross-sectional associations, but may nevertheless suggest that a relationship between job satisfaction and NPS exists although any changes in the number of pain sites induced by satisfaction with the job may not be detectable over a time period of two years.

### Methodological considerations

A strength of this study was the prospective panel design. However, generalizability may suffer from the moderate response rate (60.3% at baseline). Also, dropout was associated with skill levels and age. However, due to non-random sampling the theoretical population for which the current results may be representative remains unknown, regardless of non-response and attrition. Nevertheless, the sample did encompass 63 different organizations and a wide variety of job types. In addition to nonresponse and attrition some information was lost due to item nonresponse (as evident from Tables 2 and 3, which report N for each separate analysis). The total amount of item missing data was only 2.2%, but for three variables it was higher than 5% (job satisfaction 10.6%, mental distress 5.8, and emotional exhaustion 9.5). Due to listwise deletion missing for some analyses was higher. However, voluntary and anonymous participation leaves room for individual motivational processes to affect response rates, and one concern many participants communicated was that the comprehensive questionnaire was time-consuming to complete. Hence, response rates declined gradually throughout the questionnaire, giving little reason to suspect specific selection based on item content.

Spread and reduction of musculoskeletal pain sites may have been underestimated in the current employee sample as morbidity rates are usually lower in the working population than in the general population [45]. This may also have affected the extent to which psychological health was associated with spread of pain, as individuals that were particularly vulnerable to the health effects of psychological factors may not have been employed and represented in the study. As can be seen in Additional file 1: Table S2, the distributions of several included factors were considerably skewed with few individuals reporting high levels of potentially challenging factors.

It is important to be aware of inherent limitations of the operationalization of NPS change in the current study. The variable was based on the difference of reported number of pain sites at T1 and T2, irrespective of *which* sites were reported. This implies, for instance, that subjects reporting the same number, but different location, of pain sites at the two time points would be labeled ‘stable NPS’, whereas they may actually have experienced onset of a new pain problem as a previous problem resolved. Furthermore, subjects that reported no pain sites at T1 and were thus excluded from the main analyses may have experienced pain prior to the 4 weeks referred to in the pain questions. For instance, if a subject reported no pain sites at T1 and two pain sites at T2 this may have been spread of pain. In Additional file 1: Table S3 regressions with NPS change direction are given both with and without subjects that reported no pain sites at T1. Results were very similar, with the main difference being more statistically significant results in the higher N sample. However, the current study could not capture all possible courses of pain site development, and future studies should incorporate such distinctions in study designs and analyses to obtain more nuanced information.

All data were collected by self-report questionnaires. *Reporting bias* (e.g., due to negative affect) influencing both exposure and outcome measures may inflate associations (*common method bias (CMB))* [46]. However, if reporting bias were a major factor, one would expect indicators of negative affect, such as psychological distress, emotional exhaustion, and depression, to be core predictors of the outcome, whereas these factors were not significant predictors in the final analyses. Communicating respondent anonymity should also reduce CMB [46], and this was prioritized during the survey administration.

It is still unclear why some chronic pain disorders become widespread (e.g. fibromyalgia; [47]) while others remain localized to few musculoskeletal regions (e.g. low back pain). There may be a difference in pathophysiology of pain in different regions or there may be differences in individual predisposition or exposures that mediate spread of pain. Since the pathogenesis is obscure, one cannot know whether 2-years follow-up periods are optimal to detect predictors of pain spread. Pain spread may to a certain extent reflect normal fluctuations of pain [21] in different body regions among the healthy working population. Furthermore, mechanisms of pain spread may be different for individuals with chronic versus fluctuating pain, and from pain patients versus a healthy population. We do not know whether the respondents that reported having pain in the present study were patients with varying musculoskeletal pain disorders/chronic pain or not. The working population may not be comparable to groups with clinical pain diagnoses.

## Conclusion

A range of psychological factors, sleep problems, and pain intensities predicted both spread of pain, reduction of number of musculoskeletal pain sites, and the degree to which increase and decrease occurred over a 2-years period. Further studies are warranted, and they should aim to clarify the mechanisms involved and the causal interplay that may exist between the various risk factors.

## Additional file

Additional file 1:
**Table S1.** Descriptives for NPS and ΔNPS. **Table S2.** Descriptives for all predictors. **Table S3.**Regressions with NPS “direction of change” as outcome for the final study sample and the full sample prior to executing selection criteria. **Table S4.** Regressions with NPS “direction of change” as outcome for categorized predictors. (DOCX 36 kb)

## Abbreviations

CMB: Common method bias
HSCL: Hopkins Symptom Checklist
ISCED: International Standard Classification of Education
ISCO: International Standard Classification of Occupations
LOT-R: Revised Life Orientation Test
NPS: Number of pain sites
OR: Odds ratio
REK: Regional Commitees for Medical and Health Research Ethics (Norwegian: Regional Etisk Komitè)
T1: Time 1
T2: Time 2
