# Supplementary material for: Psychological predictors of change in the number of musculoskeletal pain sites among Norwegian employees: a prospective study
Source: BMC Musculoskelet Disord. 2017 Apr 4;18:140. doi: 10.1186/s12891-017-1503-7 (PMC5379631; doi:10.1186/s12891-017-1503-7)
Supplement: Additional file 1: — Table S1. Descriptives for NPS and ΔNPS. Table S2. Descriptives for all predictors. Table S3.Regressions with NPS “direction of change” as outcome for the final study sample and the full sample prior to executing selection criteria. Table S4. Regressions with NPS “direction of change” as outcome for categorized predictors. (DOCX 36 kb) [file 12891_2017_1503_MOESM1_ESM.docx]

| **Table S1.** Contingency table of the number of employees that reported the different combinations of number of pain sites (NPS) at T1 and T2 and descriptives for the corresponding change in the number of pain sites between T1 and T2 (Δ NPS). | | | | | | | | | | | | |
| --- | --- | --- | --- | --- | --- | --- | --- | --- | --- | --- | --- | --- |
| **All that responded at both T1 and T2 (N=4204):** | | | | | | | | | | | | |
|  | | | | | | | |  | **Change in the number of pain sites (Δ NPS)** | | | |
|  |  | **Number of pain sites** | | | | | |  | **Direction** | **N (%)** | **Δ NPS** | **N (%)** |
|  |  |  | | | | | |  |  |  |  |  |
|  |  |  |  |  |  |  |  |  | **Decrease** | 1224 (29.1) $\left\{ \begin{aligned} \\ \\ \\ \\ \end{aligned} \right.$ | -5 | 1 (0.0) |
|  |  | **NPS at T2** | | | | | |  |  |  | -4 | 12 (0.3) |
|  |  | **0** | **1** | **2** | **3** | **4** | **5** |  |  |  | -3 | 98 (2.3) |
|  | **0** | 546 | 269 | 137 | 37 | 9 | 3 |  |  |  | -2 | 293 (7.0) |
|  | **1** | 261 | 342 | 226 | 94 | 33 | 6 |  |  |  | -1 | 820 (19.5) |
| **NPS at T1** | **2** | 119 | 198 | 297 | 162 | 86 | 22 |  |  |  |  |  |
|  | **3** | 46 | 78 | 174 | 228 | 138 | 42 |  | **No change** | 1651 (39.3) | 0 | 1651 (39.3) |
|  | **4** | 10 | 31 | 63 | 132 | 136 | 65 |  |  |  |  |  |
|  | **5** | 1 | 2 | 21 | 33 | 55 | 102 |  | **Increase**  **(spread)** | 1329 (31.6) $\left\{ \begin{aligned} \\ \\ \\ \\ \end{aligned} \right.$ | 1 | 860 (20.5) |
|  |  |  |  |  |  |  |  |  |  |  | 2 | 359 (8.5) |
|  |  |  |  |  |  |  |  |  |  |  | 3 | 92 (2.2) |
|  |  |  |  |  |  |  |  |  |  |  | 4 | 15 (0.4) |
|  |  |  |  |  |  |  |  |  |  |  | 5 | 3 (0.1) |
|  |  |  |  |  |  |  |  |  |  |  |  |  |
|  | | | | | | | |  |  | | | |
| **Those that responded at both T1 and T2, and reported 1-4 pain sites at T1 (N=2989):** | | | | | | | | | | | | |
|  | | | | | | | | | | | | |
|  |  | **Number of pain sites** | | | | | |  | **Direction** | **N (%)** | **Δ NPS** | **N (%)** |
|  |  |  | | | | | |  |  |  |  |  |
|  |  |  |  |  |  |  |  |  | **Decrease** | 1112 (37.2) $\left\{ \begin{aligned} \\ \\ \\ \\ \end{aligned} \right.$ | -5 | - |
|  |  | **NPS at T2** | | | | | |  |  |  | -4 | 10 (0.3) |
|  |  | **0** | **1** | **2** | **3** | **4** | **5** |  |  |  | -3 | 77 (2.6) |
|  | **0** | - | - | - | - | - | - |  |  |  | -2 | 260 (8.7) |
|  | **1** | 261 | 342 | 226 | 94 | 33 | 6 |  |  |  | -1 | 765 (25.6) |
| **NPS at T1** | **2** | 119 | 198 | 297 | 162 | 86 | 22 |  |  |  |  |  |
|  | **3** | 46 | 78 | 174 | 228 | 138 | 42 |  | **No change** | 1003 (33.6) | 0 | 1003 (33.6) |
|  | **4** | 10 | 31 | 63 | 132 | 136 | 65 |  |  |  |  |  |
|  | **5** | - | - | - | - | - | - |  | **Increase**  **(spread)** | 874 (29.2) $\left\{ \begin{aligned} \\ \\ \\ \\ \end{aligned} \right.$ | 1 | 591 (19.8) |
|  |  |  |  |  |  |  |  |  |  |  | 2 | 222 (7.4) |
|  |  |  |  |  |  |  |  |  |  |  | 3 | 55 (1.8) |
|  |  |  |  |  |  |  |  |  |  |  | 4 | 6 (0.2) |
|  |  |  |  |  |  |  |  |  |  |  | 5 | - |

|  |  |  |  |  |  |  |  |  |  |  |  |  |
| --- | --- | --- | --- | --- | --- | --- | --- | --- | --- | --- | --- | --- |

| **Table S2.** Descriptives for psychological factors and pain intensities at T1 | | | |
| --- | --- | --- | --- |
|  | **As continuous** | **As categorical** | |
|  | **Mean (SD)** | **Response alternatives** | **N (%)** |
| **Emotional exhaustion** | 1.80 (0.64) | ‘’Never/almost never’’ | 1233 (45.6) |
|  |  | ‘’A couple of times per month’’ | 1175 (43.4) |
|  |  | ‘’Once or twice a week’’ | 227 (8.4) |
|  |  | ‘’Three to four times a week’’ | 63 (2.3) |
|  |  | ‘’’(almost) every day’’ | 8 (0.3) |
| **Mental distress** | 1.39 (0.39) | ‘’Have not experienced it’’ | 2080 (73.9) |
|  |  | ‘’Somewhat’’ | 693 (24.6) |
|  |  | ‘’Quite a lot’’ | 40 (1.4) |
|  |  | ‘’Very much’’ | 3 (0.1) |
| **Depression** (single item) | 1.30 (0.57) | ‘’Not troubled’’ | 2230 (74.9) |
|  |  | ‘’A little troubled’’ | 624 (21.0) |
|  |  | ‘’Rather intensely troubled’’ | 102 (3.4) |
|  |  | ‘’Very intensely troubled’’ | 21 (0.7) |
| **Anxiety** (single item) | 1.09 (0.35) | ‘’Not troubled’’ | 2752 (92.5) |
|  |  | ‘’A little troubled’’ | 181 (6.1) |
|  |  | ‘’Rather intensely troubled’’ | 32 (1.1) |
|  |  | ‘’Very intensely troubled’’ | 9 (0.3) |
| ***Psychological well-being*** |  |  |  |
| ‘’Having a lot of surplus’’ (single item) | 3.38 (1.21) | ’’Not at all’’ | 177 (6.2) |
|  |  | ‘’A little of the time’’ | 546 (19.0) |
|  |  | ‘’Some of the time’’ | 786 (27.3) |
|  |  | ‘’A lot of the time’’ | 823 (28.6) |
|  |  | ‘’Almost all the time’’ | 468 (16.3) |
|  |  | ‘’All the time’’ | 77 (2.7) |
| ‘’Feeling down and sad’’ (single item) | 1.75 (0.89) | ’’Not at all’’ | 1294 (45.1) |
|  |  | ‘’A little of the time’’ | 1204 (41.9) |
|  |  | ‘’Some of the time’’ | 238 (8.3) |
|  |  | ‘’A lot of the time’’ | 86 (3.0) |
|  |  | ‘’Almost all the time’’ | 27 (0.9) |
|  |  | ‘’All the time’’ | 22 (0.8) |
| **Optimism** | 3.57 (0.63) | ‘’Strongly disagree/disagree’’ | 130 (4.4) |
|  |  | ‘’Neutral’’ | 1186 (40.6) |
|  |  | ‘’Agree’’ | 1408 (48.2) |
|  |  | ‘’Strongly agree’’ | 198 (6.8) |
| **General self-efficacy** | 2.04 (0.53) | ‘’Strongly agree’’ | 436 (14.9) |
|  |  | ‘’Agree’’ | 2069 (70.7) |
|  |  | ‘’Neutral’’ | 395 (13.5) |
|  |  | ‘’Disagree’’ | 26 (0.9) |
|  |  | ‘’Strongly disagree’’ | 1 (0.0) |
| **Global job satisfaction** (single item) | 3.18 (0.61) | ‘’Very unsatisfied’’ | 24 (0.9) |
|  |  | ‘’Unsatisfied’’ | 225 (8.4) |
|  |  | ‘’Satisfied’’ | 1662 (62.2) |
|  |  | ‘’Very satisfied’’ | 761 (28.5) |
| **Sleep disturbance** | 2.15 (0.97) | ‘’None’’ | 1045 (36.5) |
|  |  | ‘’1-3 times a month’’ | 1065 (37.2) |
|  |  | ‘’1-2 times a week’’ | 517 (18.1) |
|  |  | ‘’3-5 times a week’’ | 186 (6.5) |
|  |  | ‘’6-7 times a week’’ | 51 (1.8) |
| **Tiredness** (single item) | 1.87 (0.77) | ‘’Not troubled’’ | 1021 (34.2) |
|  |  | ‘’A little troubled’’ | 1382 (46.3) |
|  |  | ‘’Rather intensely troubled’’ | 516 (17.3) |
|  |  | ‘’Very intensely troubled’’ | 65 (2.2) |
| **Restlessness** (single item) | 1.35 (0.62) | ‘’Not troubled’’ | 2143 (71.9) |
|  |  | ‘’A little troubled’’ | 644 (21.6) |
|  |  | ‘’Rather intensely troubled’’ | 169 (5.7) |
|  |  | ‘’Very intensely troubled’’ | 24 (0.8) |
| ***Pain intensities*** (single items) |  |  |  |
| Headache | 1.73 (0.80) | ‘’Not troubled’’ | 1399 (46.9) |
|  |  | ‘’A little troubled’’ | 1066 (35.7) |
|  |  | ‘’Rather intensely troubled’’ | 456 (15.3) |
|  |  | ‘’Very intensely troubled’’ | 65 (2.2) |
| Neck pain | 1.84 (0.81) | ‘’Not troubled’’ | 1181 (39.5) |
|  |  | ‘’A little troubled’’ | 1187 (39.7) |
|  |  | ‘’Rather intensely troubled’’ | 548 (18.3) |
|  |  | ‘’Very intensely troubled’’ | 73 (2.4) |
| Shoulder pain | 1.74 (0.80) | ‘’Not troubled’’ | 1380 (46.2) |
|  |  | ‘’A little troubled’’ | 1086 (36.3) |
|  |  | ‘’Rather intensely troubled’’ | 445 (14.9) |
|  |  | ‘’Very intensely troubled’’ | 78 (2.6) |
| Arm pain | 1.37 (0.68) | ‘’Not troubled’’ | 2179 (72.9) |
|  |  | ‘’A little troubled’’ | 562 (18.8) |
|  |  | ‘’Rather intensely troubled’’ | 203 (6.8) |
|  |  | ‘’Very intensely troubled’’ | 45 (1.5) |
| Leg pain | 1.36 (0.67) | ‘’Not troubled’’ | 2194 (73.4) |
|  |  | ‘’A little troubled’’ | 549 (18.4) |
|  |  | ‘’Rather intensely troubled’’ | 202 (6.8) |
|  |  | ‘’Very intensely troubled’’ | 44 (1.5) |
| Back pain | 1.72 (0.80) | ‘’Not troubled’’ | 1415 (47.3) |
|  |  | ‘’A little troubled’’ | 1089 (36.4) |
|  |  | ‘’Rather intensely troubled’’ | 406 (13.6) |
|  |  | ‘’Very intensely troubled’’ | 79 (2.6) |
| Note: For multi-item factors distributions are for *categorized* scores based on the means of multiple items, and for single item factors distributions are for raw scores. | | | |

| **Table S3.** Multinomial logistic regressions comparing employees reporting the same number of pain sites (NPS) at T1 and T2 with those experiencing decrease and increase (spread). Analyses were also run without subjects reporting no or five pain sites at T1^a^. All regressions were run separately, adjusted for sex, age category, skill level, and number of pain sites at T1. | | | | | | | | | | |
| --- | --- | --- | --- | --- | --- | --- | --- | --- | --- | --- |
|  | **All employees** | | | | | **Employees that reported 1-4 pain sites at T1** | | | | |
|  |  | **Decrease NPS** | | **Increase NPS**  **(spread + onset)** | |  | **Decrease NPS** | | **Increase NPS (spread)** | |
| **T1 Predictor** | **N** | **OR** | **95% CI** | **OR** | **95% CI** | **N** | **OR** | **95% CI** | **OR** | **95% CI** |
| **Emotional exhaustion** | 3686 | 0.80 | [0.70-0.93]** | 1.38 | [1.21-1.58]** | 2644 | 0.81 | [0.69-0.95]* | 1.34 | [1.15-1.57]** |
| **Mental distress** | 3847 | 0.65 | [0.51-0.81]** | 1.65 | [1.33-2.03]** | 2752 | 0.69 | [0.54-0.89]** | 1.64 | [1.28-2.10]** |
| **Depression (single item)** | 4081 | 0.91 | [0.79-1.06] | 1.22 | [1.05-1.40]** | 2912 | 0.90 | [0.76-1.05] | 1.16 | [0.99-1.37] |
| **Anxiety (single item)** | 4078 | 0.78 | [0.62-0.99]* | 1.15 | [0.92-1.42] | 2909 | 0.86 | [0.66-1.11] | 1.09 | [0.84-1.41] |
| ***Psychological well-being*** |  |  |  |  |  |  |  |  |  |  |
| ”Had a lot of surplus” | 3932 | 1.01 | [0.94-1.08] | 0.85 | [0.80-0.91]** | 2814 | 1.02 | [0.95-1.10] | 0.87 | [0.80-0.95]** |
| ”Felt down and sad” | 3925 | 0.97 | [0.88-1.06] | 1.19 | [1.09-1.29]** | 2808 | 0.98 | [0.88-1.09] | 1.18 | [1.06-1.31]** |
| **Dispositional optimism** | 4008 | 1.03 | [0.90-1.17] | 0.87 | [0.77-0.98]* | 2858 | 1.08 | [0.94-1.25] | 0.94 | [0.81-1.09] |
| **General self-efficacy** | 4017 | 1.04 | [0.89-1.21] | 1.09 | [0.95-1.25] | 2862 | 0.97 | [0.82-1.14] | 1.05 | [0.88-1.26] |
| **Job satisfaction** | 3658 | 1.00 | [0.87-1.15] | 0.86 | [0.75-0.98]* | 2608 | 1.05 | [0.90-1.22] | 0.97 | [0.82-1.15] |
| **Sleep disturbance** | 3922 | 0.87 | [0.80-0.95]** | 1.22 | [1.12-1.32]** | 2801 | 0.87 | [0.79-0.96]* | 1.19 | [1.07-1.31]** |
| **Tiredness** | 4090 | 0.86 | [0.77-0.96]** | 1.34 | [1.20-1.48]** | 2919 | 0.82 | [0.73-0.93]** | 1.24 | [1.09-1.40]** |
| **Restlessness** | 4087 | 0.79 | [0.69-0.90]** | 1.10 | [0.97-1.25] | 2915 | 0.80 | [0.69-0.92]** | 1.08 | [0.93-1.25] |
| ***Pain intensity*** |  |  |  |  |  |  |  |  |  |  |
| Headache | 4096 | 0.83 | [0.74-0.92]** | 1.24 | [1.12-1.38]** | 2921 | 0.81 | [0.72-0.92]** | 1.17 | [1.04-1.32]* |
| Neck pain | 4100 | 0.62 | [0.54-0.70]** | 1.05 | [0.92-1.19] | 2897 | 0.64 | [0.56-0.73]** | 1.02 | [0.90-1.17] |
| Shoulder pain | 4100 | 0.71 | [0.62-0.81]** | 0.96 | [0.84-1.09] | 2924 | 0.78 | [0.68-0.89]** | 0.98 | [0.85-1.12] |
| Leg pain | 4100 | 0.89 | [0.79-1.01] | 0.89 | [0.78-1.03] | 2924 | 1.06 | [0.93-1.21] | 1.03 | [0.88-1.19] |
| Back pain | 4100 | 0.84 | [0.75-0.95]** | 1.06 | [0.94-1.19] | 2924 | 0.85 | [0.76-0.96]* | 1.06 | [0.93-1.20] |
| Arm pain | 4100 | 0.87 | [0.77-0.99]* | 0.83 | [0.71-0.97]* | 2924 | 1.09 | [0.95-1.25] | 0.97 | [0.83-1.14] |
| **Number of pain sites** | 4100 | 1.70 | [1.61-1.80]** | 0.81 | [0.76-0.85]** | 2924 | 1.33 | [1.23-1.45]** | 0.79 | [0.72-0.87]** |

* p< 0.05, ** p< 0.01

^a^ Subjects reporting no pain sites at T1 but at least one pain site at T2 may be experiencing *onset* rather than *spread* of pain. Subjects reporting five pain sites at T1 could not experience further spread of pain.

| ***Table S4.*** Categorized predictors: Separate multinomial logistic regressions relating psychological factors and pain intensities at T1 to decrease and increase of the number of pain sites (NPS) between T1 and T2, with stability of NPS (T1 NPS=T2 NPS) as reference. | | | | | | |
| --- | --- | --- | --- | --- | --- | --- |
|  |  | **Decrease NPS** | | **Increase NPS** | |  |
|  | **Category** | **OR** | **95% CI** | **OR** | **95% CI** | **LRT^a^** |
| **Emotional exhaustion** | 1 | ref | - | ref | - | 1.00 |
|  | 2 | 0.84 | [0.69-1.03] | 1.13 | [0.91-1.39] |  |
|  | 3 | 0.61 | [0.42-0.89]** | 1.58 | [1.10-2.28]** |  |
|  | 4 | 0.63 | [0.33-1.20] | 1.67 | [0.87-3.18] |  |
|  | 5 | 1.23 | [0.11-13.76] | 6.57 | [0.71-60.74] |  |
| **Mental distress (HSCL)** | 1 | ref | - | ref | - | 1.00 |
|  | 2 | 0.77 | [0.61-0.96]* | 1.54 | [1.23-1.93]*** |  |
|  | 3/4 | 0.77 | [0.36-1.62] | 1.35 | [0.62-2.95] |  |
| **Depression (single item)** | 1 | ref | - | ref | - | 0.63 |
|  | 2 | 0.90 | [0.72-1.12] | 1.24 | [0.99-1.56] |  |
|  | 3 | 0.88 | [0.53-1.46] | 1.50 | [0.89-2.52] |  |
|  | 4 | 0.57 | [0.21-1.55] | 0.73 | [0.24-2.24] |  |
| **Anxiety (single item)** | 1 |  |  |  |  | 0.56 |
|  | 2 | 0.90 | [0.63-1.30] | 1.09 | [0.74-1.61] |  |
|  | 3 | 0.42 | [0.16-1.06] | 1.13 | [0.50-2.56] |  |
|  | 4 | 1.50 | [0.27-8.37] | 1.60 | [0.26-9.91] |  |
| **Psychological well-being** |  |  |  |  |  |  |
| *“Had a lot of surplus”* | 1 | ref | - | ref | - | 0.20 |
|  | 2 | 0.67 | [0.44-1.02] | 0.75 | [0.48-1.17] |  |
|  | 3 | 0.79 | [0.52-1.19] | 0.70 | [0.45-1.08] |  |
|  | 4 | 0.87 | [0.58-1.32] | 0.65 | [0.42-1.01]* |  |
|  | 5 | 0.78 | [0.50-1.21] | 0.43 | [0.27-0.69]*** |  |
|  | 6 | 0.91 | [0.46-1.80] | 0.73 | [0.37-1.47] |  |
| *“Felt down and sad”* | 1 | ref | - | ref | - | 0.20 |
|  | 2 | 0.90 | [0.75-1.10] | 1.23 | [1.00-1.51]* |  |
|  | 3 | 1.05 | [0.73-1.52] | 2.08 | [1.44-3.01]*** |  |
|  | 4 | 0.79 | [0.47-1.34] | 1.13 | [0.65-1.99] |  |
|  | 5 | 1.30 | [0.50-3.38] | 1.64 | [0.58-4.64] |  |
|  | 6 | 1.04 | [0.34-3.15] | 1.69 | [0.58-4.98] |  |
| **Dispositional optimism** | 1 | ref | - | ref | - | 0.26 |
|  | 2 | 1.01 | [0.65-1.59] | 0.94 | [0.59-1.48] |  |
|  | 3 | 1.02 | [0.65-1.58] | 0.83 | [0.52-1.30] |  |
|  | 4 | 1.56 | [0.90-2.70] | 0.97 | [0.54-1.74] |  |
| **General self-efficacy** | 1 | ref | - | ref | - | 0.10 |
|  | 2 | 0.79 | [0.61-1.01] | 0.90 | [0.68-1.18] |  |
|  | 3 | 0.98 | [0.70-1.37] | 1.24 | [0.87-1.78] |  |
|  | 4 | 0.54 | [0.20-1.41] | 0.87 | [0.33-2.31] |  |
|  | 5 | na | na | na | na |  |
| **Job satisfaction (single item)** | 1 | ref | - | ref | - | 0.10 |
|  | 2 | 0.54 | [0.20-1.50] | 1.53 | [0.36-6.43] |  |
|  | 3 | 0.50 | [0.19-1.33] | 1.53 | [0.38-6.21] |  |
|  | 4 | 0.60 | [0.22-1.61] | 1.44 | [0.35-5.90] |  |
| **Sleep disturbance** | 1 | ref | - | ref | - | 0.43 |
|  | 2 | 0.87 | [0.71-1.08] | 1.45 | [1.15-1.81]*** |  |
|  | 3 | 0.72 | [0.56-0.94]** | 1.46 | [1.11-1.92]** |  |
|  | 4 | 0.71 | [0.48-1.06] | 1.90 | [1.27-2.85]*** |  |
|  | 5 | 0.69 | [0.35-1.36] | 1.21 | [0.58-2.53] |  |
| **Tiredness** | 1 | ref | - | ref | - | 0.84 |
|  | 2 | 0.89 | [0.73-1.09] | 1.33 | [1.07-1.65]** |  |
|  | 3 | 0.68 | [0.52-0.89]** | 1.57 | [1.19-2.08]*** |  |
|  | 4 | 0.50 | [0.26-0.96]* | 1.59 | [0.85-2.99] |  |
| **Restlessness** | 1 | ref | - | ref | - | 0.41 |
|  | 2 | 0.72 | [0.58-0.90]*** | 1.01 | [0.80-1.26] |  |
|  | 3 | 0.73 | [0.49-1.09] | 1.44 | [0.97-2.14] |  |
|  | 4 | 0.59 | [0.23-1.52] | 0.74 | [0.26-2.07] |  |
| **Pain intensities** |  |  |  |  |  |  |
| Headache | 1 | ref | - | ref | - | **0.01*** |
|  | 2 | 0.64 | [0.52-0.78]*** | 1.20 | [0.97-1.49] |  |
|  | 3 | 0.69 | [0.52-0.90]** | 1.31 | [0.98-1.74] |  |
|  | 4 | 0.77 | [0.40-1.47] | 1.98 | [1.05-3.74]* |  |
| Shoulder pain | 1 | ref | - | ref | - | **0.00***** |
|  | 2 | 0.93 | [0.74-1.17] | 0.78 | [0.61-1.00]* |  |
|  | 3 | 0.60 | [0.44-0.81]*** | 1.01 | [0.73-1.39] |  |
|  | 4 | 0.48 | [0.26-0.86]** | 0.94 | [0.51-1.71] |  |
| Neck pain | 1 | ref | - | ref | - | 0.13 |
|  | 2 | 0.66 | [0.53-0.82]*** | 0.87 | [0.69-1.10] |  |
|  | 3 | 0.37 | [0.27-0.50]*** | 0.99 | [0.74-1.34] |  |
|  | 4 | 0.38 | [0.20-0.71]*** | 1.45 | [0.77-2.72] |  |
| Leg pain | 1 | ref | - | ref | - | **0.00***** |
|  | 2 | 1.67 | [1.31-2.12]*** | 1.34 | [1.02-1.75]* |  |
|  | 3 | 0.95 | [0.67-1.35] | 0.98 | [0.67-1.45] |  |
|  | 4 | 0.61 | [0.29-1.25] | 0.72 | [0.33-1.56] |  |
| Back pain | 1 | ref | - | ref | - | **0.00***** |
|  | 2 | 1.23 | [1.00-1.51]* | 1.01 | [0.81-1.26] |  |
|  | 3 | 0.67 | [0.50-0.90]** | 1.18 | [0.88-1.59] |  |
|  | 4 | 0.48 | [0.27-0.86]** | 1.11 | [0.63-1.94] |  |
| Arm pain | 1 | ref | - | ref | - | **0.00***** |
|  | 2 | 1.62 | [1.28-2.06]*** | 1.10 | [0.84-1.45] |  |
|  | 3 | 0.93 | [0.65-1.32] | 0.96 | [0.64-1.42] |  |
|  | 4 | 1.00 | [0.50-1.97] | 0.66 | [0.27-1.64] |  |
| **T1 Number of pain sites** | 1 | ref | - | ref | - | 0.53 |
|  | 2 | 1.40 | [1.12-1.77]*** | 0.83 | [0.67-1.05] |  |
|  | 3 | 1.79 | [1.41-2.28]*** | 0.71 | [0.55-0.92]** |  |
|  | 4 | 2.36 | [1.80-3.10]*** | 0.43 | [0.31-0.60]*** |  |
| *p<0.05, **p<0.01, ***p<0.001  ^a^ Likelihood ratio tests (LRTs) were used to determine whether a categorical predictor improved the model over the corresponding model with a linear predictor. A statistically significant test result suggests that a categorical predictor adds information to the model.  na: na applicable, insufficient information | | | | | | |
